# Forecasting *in vivo* pharmacokinetics of metformin HCl floating beads using Gastroplus® PBPK

**DOI:** 10.12688/f1000research.159438.2

**Published:** 2025-09-09

**Authors:** Sura Zuhair Mahmood, Nora Zawar Yousif, Masar Basim Mohsin Mohamed

**Affiliations:** 1 Pharmaceutics Department, Pharmacy College, Mustansiriyah University, Baghdad, Iraq

**Keywords:** Ionic gelation; Na alginate, gastroretentive, effervescent system, multiple units, in silico

## Abstract

**Background:**

Diabetes mellitus type II is expected to impact a large population worldwide. Among the available therapeutic options, Metformin hydrochloride is a key medication, particularly for those who cannot effectively manage the condition through changes in diet and lifestyle alone. This research aimed to predict their *in vivo* parameters from an *in vitro* release study by developing a physiologically based pharmacokinetic (PBPK) model by using Gastroplus ^®^ software.

**Methods:**

Sodium alginate-based MH floating beads were prepared by dissolving different concentrations of sodium alginate in deionized water, incorporating MH (1 g) and calcium carbonate (1.5 mg) as a gas-forming agent, and mixing at 200 rpm. The air-free dispersion, achieved through 30 minutes of sonication, was dropped into a 5% w/v calcium chloride solution containing 5% v/v isopropyl alcohol via a syringe for cross-linking and bead formation. Beads were cured in the solution for 30 minutes to enhance mechanical strength, then filtered, washed, and air-dried for 24 hours, ensuring uniformity and stability for controlled drug delivery and the prepared beads were evaluated for their entrapment efficiency %, morphology, floating property and *in vitro* release Ultimately, using Gastroplus ^®^ software, to predict the pharmacokinetic profile of *in vitro* release results.

**Results:**

Entrapment efficiency exhibited acceptable values, and the beads were smooth, and rounded in shape for all formulations. The beads remained afloat during the release study; the release study revealed that F1 to F5 showed asymptotic slow-release, while F6 and F7 gave shorter release times. The prediction of absorption indicated highest MH absorption was in the jejunum, then the duodenum.

**Conclusion:**

The prepared Beads had promising pharmacokinetic parameters and C max was close to MH modified released tablet.

## Introduction

According to International Diabetes Federation (IDF) in 2021, approximately 537 million individuals worldwide about (10.5%) of the global population, especially those aged 20–79 years are living with diabetes, this number is growing to be increased within a few upcoming decades. ^1^ Several protocols guide to treatment of diabetes mellitus II, and Metformin hydrochloride (MH) is one of the medicines used in the treatment, especially for those who cannot control the illness with nutrition and lifestyle alone. Many studies also covered the use of MH in obesity treatment, as bulky cohort studies showed the significant weight loss associated with MH therapy. ^2^ MH has a favourable clinical response with few negative consequences since MH does not cause hypoglycemia at any tolerable dose. Some challenges arose upon MH prolonged therapy, such as the high dose of 0.5 to 3 g per day. Also, the MH biological half-life is 1.5-3 h with low bioavailability. Moreover, MH was correlated to the drugs that demonstrated a narrow absorption window, as the proximal small intestine is the primary site of MH absorption. Hence, developing controlled-release dosage forms of MH by selecting the gastroretentive technology was the solution to embrace the previous issues. ^3^

Numerous approaches and dosage forms enable prolonged residence in the stomach; even so, floating bead units are considered for gastric retention in this study. Beads are unique spherical microcapsules serving drug encapsulation within the bead’s core that permits slow release. Multiple floating bead units accomplish the goal of developing gastroretentive drug delivery systems by sustaining drug release and prolonging stomach residence. Compared to single unit preparations, multiparticulate unit systems have significant merits, notably uniform dispersion in the gastrointestinal tract (GIT), unvarying drug absorption, reduced inter- and intra-individual variability, less potential for dose dumping, and improved flow property. ^4, 5^ Former studies investigated multiple unit floating beads, such as Yasir Mohd et al prepared combined Na alginate and pectin multiple unit floating beads to prolong the MH release for up to 12 hr by emulsification gelation technique. ^6^ Also, Singh A. et al developed metformin floating microsphere using Eudragit RS 100 controlling polymer, thus providing a sustained release pattern up to 12 hr. ^7^ Byungsuk K. et al, a sustained-release non-effervescent floating matrix tablet was prepared using a simple and efficient direct compression of spray-dried granules containing metformin hydrochloride to prolong the release of MH for 24 hr. ^8^ Recently, MH was prepared as a core-shell hydrogel and nanoparticles to retain and sustain the release. ^9, 10^

The goal of this research mainly involves the use of Gastroplus ^®^ software (version 9.8.2, SimulationPlus Inc., Lancaster, CA, USA) to obtain a model for MH that helped to predict pharmacokinetic parameters for the *in vitro* release study for the first time in MH multiple unit floating beads. The same goal was used to gain *in vivo* prediction for the *in vitro* release using Gastroplus ^®^ software for metoclopramide HCl ^11^ and sildenafil. ^12^ This research marks our initial utilization of GastroPlus ^®^ software to predict *in vivo* pharmacokinetic parameters from *in vitro* data for metformin bead formulations, while previous studies focused on MH simulation using Gastroplus ^®^ software for further scopes, including expecting the outcome of 1000 mg tablet clinically. ^13^ Also, a PBPK model of 850 mg metformin HCl for healthy and renal impaired sick patients was used to investigate the parameters that described renal dysfunction. ^14^^,^^15^ Moreover, Dahan *et al* improved *in silico* metformin model to study absorption after the shortening of gastrointestinal segments by bypass surgery, which is highly recommended for obese type II diabetic patients. ^16^ The PBPK model depends on several equations embedded within the Gastroplus that control the dissolution, absorption, and other physiological processes within the body of humans or animals. Building PBPK models needs many inputs that are related to the physical properties of the compounds, in addition to the information that specifies the drug metabolism and permeability. The output of this process is the ability to predict the *in vivo* data of the interested compound. ^17^

## Methods

### Materials

Na alginate was purchased from Fine chem limited, and the Metformin hydrochloride was kindly gifted from Samara Drug Industry (Iraq). Calcium chloride (99.89%), Calcium carbonate, and isopropyl alcohol were purchased from BDH, Ind., England (as listed in Table 1).

**Table 1.** List of reagents.

Reagent	Amount	Catalogue number
Calicum carbonate	1 kg	22313.294
Calicium chloride	250 gm	22300.233
Isopropyl alcohol	500 ml	0918-500 ML

### Methods

***Formulation of floating beads***

Different concentrations of sodium alginate solution were prepared for use in the MH floating beads formulation, as shown in Table 2. Each solution was made by using a magnetic stirrer to dissolve the appropriate amount of Na alginate in 100 ml of deionized water with gentle strirring. An alginate solution containing 1 gm MH and 1.5 mg calcium carbonate as a gas-forming agent was mixed well by stirring at a constant speed 200 rpm at room temperature. All remaining air bubbles in the dispersion were removed using a sonicator for 30 minutes. The resulting dispersion was introduced to a 100 ml solution of 5% (w/v) calcium chloride (fused) in isopropyl alcohol 5% v/v (isopropyl alcohol is used as dispersing agent and cross-linking agent, so might be these properties play an important role in uniform bead formation) at room temperature via a 23-gauge syringe needle. ^5^ The mechanical strength of the beads was improved by leaving them in the curing solution for 30 minutes. The beads went through many rounds of filtration and washing with water, after which they were air-dried for 24 hours. ^18^

**Table 2.** Composition of MH alginate floating Beads.

Formulation code	Na alginate ( *gm*)
**F1**	1
**F2**	1.5
**F3**	2
**F4**	2.5
**F5**	3
**F6**	0.25
**F7**	0.5

* Each formulation contains MH, CaCO _3_, and CaCl _2_ equal to 1 gm, 1.5 gm, 5 gm, and respectively.

***Percentage of yield***

The percentage of yield depends on the ratio of polymer and gas forming agent, which the following formula can calculate ^19^:

%Yeild=(Calculated yield/Theoretical yield(Polymer+Drug)×100

***Percentage of entrapment efficiency %***

Fifty milligrams of MH beads were crushed and added to a 100 ml solution of 0.1 *N* HCl pH 1.2 to be filtered after 24 hr of incubation with constant stirring; then MH was analyzed using a UV/VIS spectrophotometer (UV Spectrophotometer, 1601, Shimadzu, Japan) at 233 nm, the maximum wavelength and dilution of the filtrate with 0.1N HCl pH 1.2. The following equation was used to calculate the entrapment efficiency. The individual batch should be examined for drug content in triplicate. ^20^

Entrapment efficiency%=(Actual drug content/theoretical drug content)×100

***Floating properties (Buoyancy lag time and duration of buoyancy)***

Buoyancy lag time starts from the beads’ introduction into the medium until their buoyancy to the surface of the dissolution vessel, and the buoyancy duration means the time for which the beads constantly float on the surface of the medium. Both tests were performed during the *in vitro* release study by visual observation. Buoyancy lag time was determined by weighing equivalent to 500 mg of MH beads and placing them into a dissolution vessel paddle type containing 900 ml of 0.1 N HCl, pH 1.2 at 37 ± 0.5°C at 50 rpm. ^21^ All the determinations were conducted in triplicate.

***In vitro* release study**

*In vitro*, release study investigations were carried out using a USP Dissolution apparatus Type II. The dissolution medium was 900 ml of simulated gastric fluid 0.1 N HCl (pH 1.2) at 37±0.5 ^o^C. A sufficient quantity of beads equivalent to 500 mg of MH was placed in the dissolution medium. The paddle speed was limited to 50 rpm, and 5 ml samples were taken and replaced with fresh medium hourly for up to 12 hr. The withdrawal samples were analyzed using a UV/VIS spectrophotometer (UV Spectrophotometer, 1601, Shimadzu, Japan) at 233 nm.

***Morphological examination***

The selected beads were viewed under a Field Emission Scanning Electron Microscope (FESEM) (Inspect ^TM^ F50, FEI company, USA). The surface of the beads and their cross sections were coated with gold-palladium under an argon atmosphere using a gold sputter module in a high vacuum evaporator.

***Fourier Transmittance Infrared (FTIR)***

Shimadzu- 8300, Japan FT-IR spectroscopy was utilized for Na alginate, MH as powder, and the selected drug-loaded beads were milled and then put in a KBr press. The spectra were taken from 4000 to 400 cm ^−1^.

***In silico modeling for metformin absorption***

The Gastroplus ^®^ software (version 9.8.2, SimulationPlus Inc., Lancaster, CA, USA ‘ SLP Cloud Access /acadmic access’ helped construct an MH model to gain a prediction of *in vivo* pharmacokinetic parameters for *in vitro* multiple unit beads release data. Table 3 presents input parameters related to MH physicochemical and pharmacokinetic properties taken from the literature and/or *in silico* estimated. The constructed model relies on divided segments of the gastrointestinal tract according to Advanced Compartmental Absorption and Transient (ACAT) model composed of the following: stomach, duodenum, jejunum 1 and 2, ileum 1-3, caecum and ascending colon. The model construction depended on 500 mg of each intravenous bolus and immediate–release oral tablet of MH. The GetData Digitizer version 2.26.0.2 software was used to extract metformin 500 mg of intravenous and oral immediate-release tablet data of healthy individuals aged 42 and weighing 63.4 kg, which was the base of the PBPK. ^22^ The clearance values shown in Table 3 were obtained by applying the PKPlus software module to 500 mg intravenous metformin and clarified a three-compartmental model.

**Table 3.** Input parameters used in n the compound window of GastroPlus as (*) and (**) values were predicted by ADMET predictor in addition to PKPlus in Gastroplus® software (version 9.8.2, Simulation Plus, Inc., Lancaster, CA, USA), respectively.

Parameter	Input	Reference
Dose	500 mg	
Molecular weight	129.17 g/mol	*
Dosage form	Immediate release tablet	
Log P at pH -1	-0.82	*
Solubility at pH 12.9	134.78 mg/ml	. ^14^
Diffusion Coefficient	1.14 x 10 ^−5^ cm ^2^/s	*
Drug particle density	1.2 g/ml	*
Effective permeability	0.032 cm/s x 10 ^−5^	. ^28^
Clearance ^a^	30.828 L/h	*
Clearance ^a^	0.48625 L/h/kg	*
K12 ^b^	0.70212 1/h	**
K21 ^b^	0.76947 1/h	**
K13 ^b^	0.21387 1/h	**
K 31 ^b^	0.001192 1/h	**
V2 ^c^	0.4317 L/kg	**
V3 ^c^	84.893 L/kg	**
OCT1 ^d^ km ^g^	1.47 mM	. ^24^
OCT1 ^d^ Vmax ^h^	396 pmol/min/mg protein	. ^24^
OCT2 ^d^ km	0.99 mM	. ^24^
OCT2 ^d^ Vmax	1444 pmol/min/mg protein	. ^24^
MATE1 ^e^ km	0.78 mM	. ^29^
MATE1 ^e^ Vmax	4.46 nmol/min/mg protein	. ^15^
MATE-K2 ^e^ km	1.98 mM	. ^29^
MATE-K2 ^e^ Vmax	1.69 nmol/min/mg protein	. ^15^
PMAT ^f^ km	1.32 mM	. ^15^
PMAT ^f^ Vmax	27 pmol/min/mg protein	. ^26^

^a^ Cl donates as clearance.

^b^ k12, k 21, k13 and k31 donate as elimination phase constants of 3 compartmental model.

^c^ V2 and V3 donates volume of distribution.

^d^ OCT 1 and OCT 2 denote organic cation transporters.

^e^ MATE1 and MATE2-K donate the multidrug and toxin extrusions.

^f^ PMAT donates Plasma Membrane Monoamine Transporter.

^g^ km donate the measurements of the affinity of the substrate for the transporter.

^h^ Vmax denote maximum rates of metabolism.

As MH was classified III according to the Biopharmaceutical classification system (BCS), which is water soluble with low permeability; ^23^ hence, the PBPK model was set as permeability limited.

MH was reported as a substrate for the organic cation transporters (OCTs), which are influx transporters starting with OCT1, primarily found in the human liver, and OCT2 transporters are located mainly in the kidney. Thus, these two transporters were included in the PBPK model. Nonetheless, the OCT3 were not included the simulation of the MH model as they showed low affinity to MH and its expression in the region of the human small intestine. ^24^ Efflux transporters the multidrug and toxin extrusions (MATE1 and MATE2-K) were MH substrate as the MATE1 expression is mainly in the liver and kidney cells membrane. The kidney cell’s membrane is the prominent place of MATE2-K. ^25^ In addition, Plasma Membrane Monoamine Transporter (PMAT) identified affinity for MH uptake and expressed in the human small intestine. All the km and Vmax values of the transporters are presented in Table 3.

In the prediction process after model building, many inputs were added, starting with beads *in vitro* release results were entered with a change of gastric physiological emptying time to the floating beads time, and the (controlled release) CR gastric was chosen as the input of the dosage form.

The predicted model verification was by using the error percentage equation ^26, 27^:

$$\%\mathrm{PE}=\frac{\left(\text{Observed}-\text{calculated}\;\right)}{\text{Observed}}\times 100$$

## Results and Discussion

### Beads formulation

The ionotropic gelation method was effective in preparing the multiparticulate bead with all conditions used, such as stirring rate, the temperature of preparation and the percentage of calcium chloride solution. All the formulations in Table 2 successfully and visually showed beads and collected to be stored for further investigations.

### Percentage of yield

All beads were subjected to the yield percentage test to assess the effect of the bead’s content, primarily the Na alginate, and the results are shown in Table 4. The results found that the increase in Na alginate concentration (F6, F7, F1-F4) increased the percentage yield of produced beads. This finding was similar to Singh *et al* finding of floating microspheres of famotidine. ^30^ This means the increased Na alginate amount helped in better bead formulations, as viscosity enhancement may result in improved crosslinking and consequently efficient bead formation. ^31^ Except for F5 the percentage of yield was diminished, this may be related to the formation of a thicker solution that acts as a barrier to form beads easily. The same inference was observed by previous researcher Tønnesen HH. ^32^

**Table 4.** The percentage of yield and entrapment efficiency of MH beads.

Code of beads formulation	%yield	Entrapment efficiency
F1	73%± 0.56	60%±0.011
F2	75%±0.63	68%±0.065
F3	77%±0.11	70%±0.045
F4	90%±0.45	79%±0.023
F5	85%±0.21	69%±0.105
F6	70%±0.51	75%±0.230
F7	72%±0.54	80%±0.124

### Percentage of entrapment efficiency %

An important consideration when designing new bead formulations is how effectively they hold the drug. Table 4 shows that all formulations (F1-F7) showed acceptable entrapment efficiency percent %, referring that Na alginate polymer concentrations are satisfactory for enclosing the drug in bead formulations, a similar finding was noticed in Abbas *et al.*’s work of enalapril floating microspheres as a controlled release dosage form. ^33^ Despite the small differences in entrapment efficiency values of all bead formulations, this might be attributed to the layers of Na alginate that were formed, which led to a decrease in the inbox of MH.

### Floating properties (buoyancy lag time and duration of buoyancy)

It was essential for the current study to investigate the floating property, which was one of the study goals. All the formulations of beads floated without delay. This could be explained by the fact that an increase in Na alginate concentration leads to a robust matrix of the strong gel and increases diffusion path length for the gas, thus effectively entrapping generated gas bubbles, ^34^ except F6, which contains the lowest amount of the polymer (0.250 gm) consequently, a weaker gel matrix can be formed resulting in a lag time of 30 min, however, collectively the formulas remained floated for the whole release study time. This finding is comparable to what was observed with floating alginate beads of curcumin. ^35^

### *In vitro* drug release

**Figure 1.**
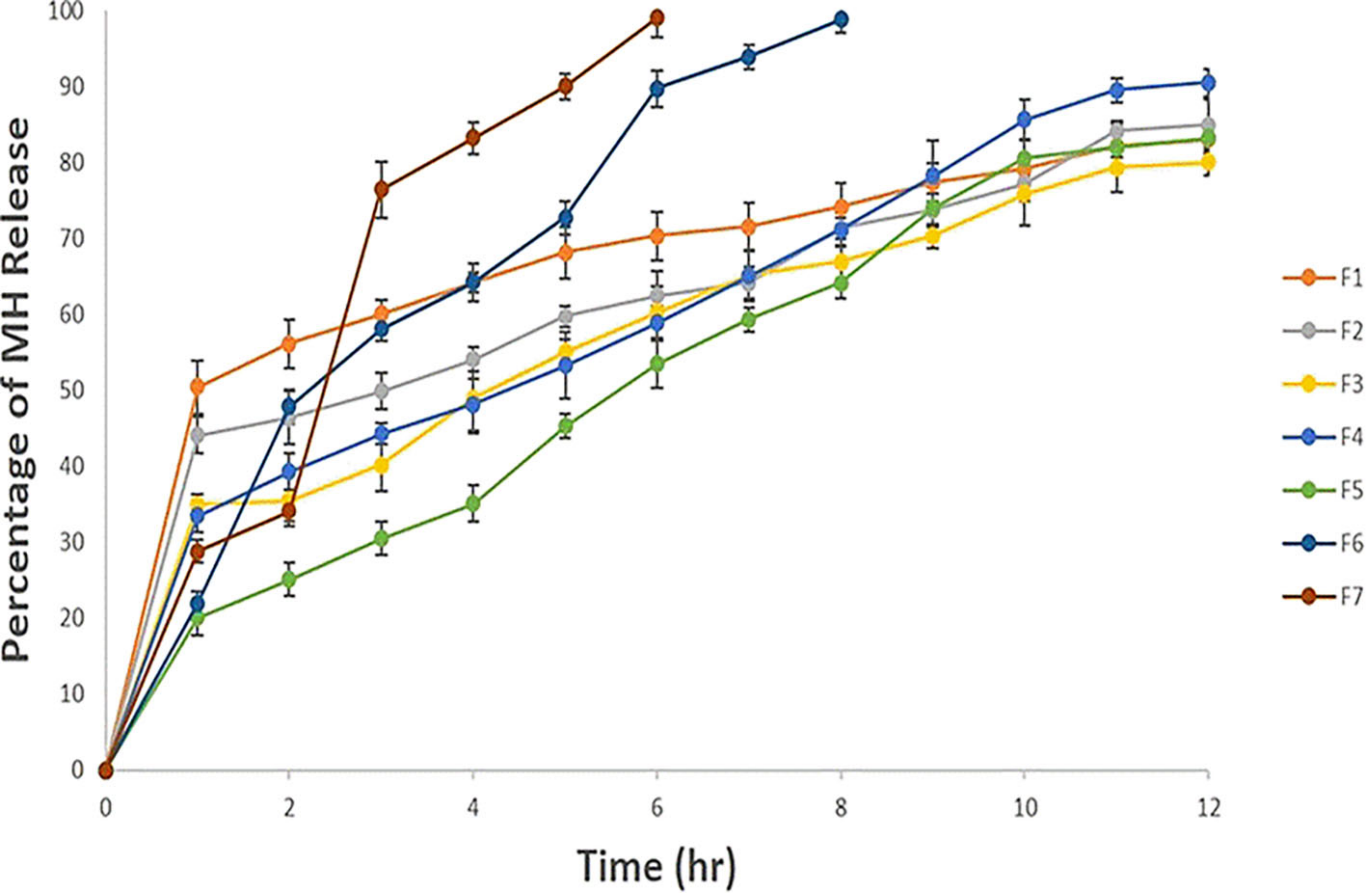
The *in vitro* release study in 0.1 N HCl pH 1.2 as each release profile represents an average triplicate ±SD for each bead formulation.

The MH release from beads was required to compare the drug release profile from different bead formulations, and the results are illustrated in Figure 1. The MH release was higher with a lower Na alginate amount, as in F6 and F7. The gelation process is based on the formation of tights between the guluronic acid residues in Na alginate and CaCl _2,_ which might increase as the Na alginate concentration increases, thus resulting in prolonged drug release, as was noticed with the rest of the prepared formulations, this observation was justified by Mandal *et al* either. ^36^ Also, it was clear from Figure 1 that the bead formulations F1, F2, F3, F4 and F5 exhibited very similar release profiles and around 80 % MH was released gradually after 12 hours, while F6 and F7 released the MH after 6 hr and 8 hr, respectively.

### Morphological examination

**Figure 2.**
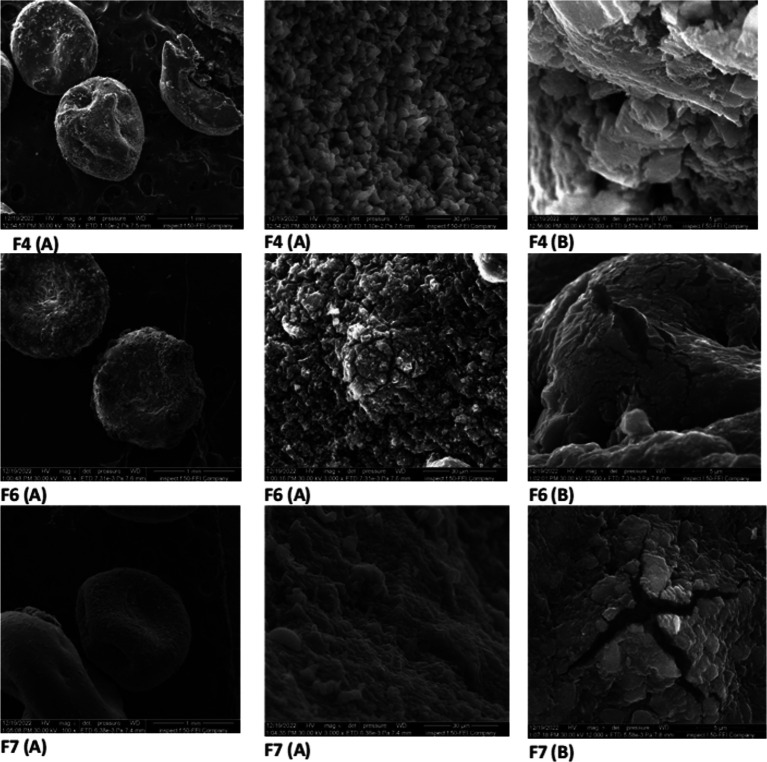
(A) Field emission scanning electron microscopy (FESEM) micrographs for the surface view as the magnification bars are 1mm and 30 μm for F4, F6 and F7 bead formulations, (B) (FESEM) microimages for cross-sectional view, magnification bar is 5 μm for F4, F6 and F7 bead formulations.

The F4 (representative beads of F1, F2 and F3), F6 and F7 were selected for morphological examination and FTIR. The morphological investigation by FESEM gives an idea about the shape and the surfaces of the formed bead. Figure 2 reveals that the beads are rounded in shape, suggesting that the concentrations of the polymer were satisfactory for bead formation, the beads apparently exhibit smoother surfaces as the concentration of Na alginate increases as in F4 in comparison to F6 and F7; this may be due to the higher density of crosslinked polymer matrices at the surface. The inside of the beads, as it appears in cross-sectional view, didn’t show distinguished morphology. A similar finding was noticed by Voo Wang-Ping *et al*. ^37^ Visually, the bead diameter of F4, F6 and F7 was around 1mm, as exhibited in the first left column of Figure 2. The same value of the diameter (1mm) was obtained in a different study prepared alginate beads. ^38^

### Fourier transmittance infrared (FTIR)

**Figure 3.**
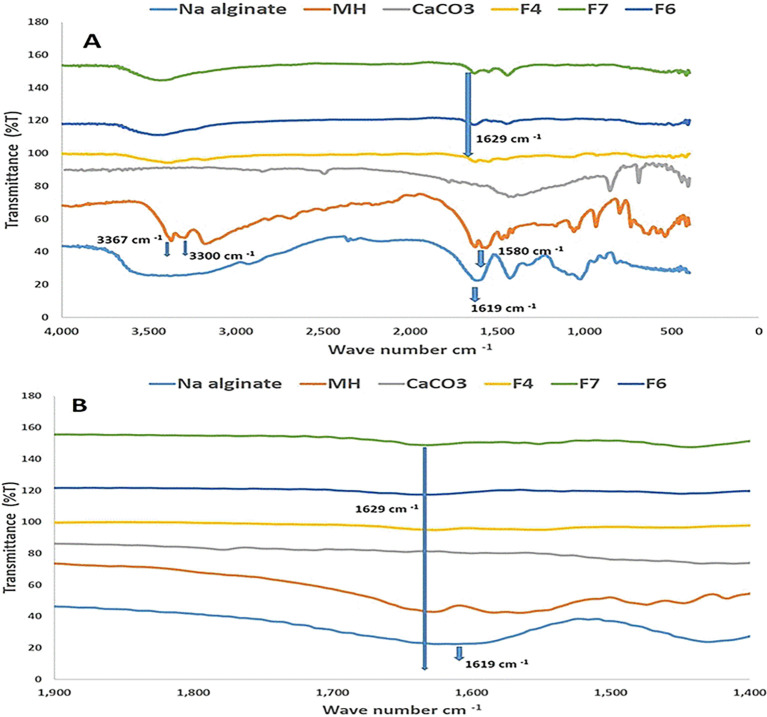
The whole FTIR spectrograms in Figure A represent the full range spectrograms for F4, F6, F7, pure drug, Na alginate, and CaCO _3_; Figure B indicates carbonyl group shifting in F4, F6, and F7.

To understand the interactions between molecules formulating beads, FTIR was applied. The FTIR spectrogram of MH, as presented in Figure 3(A), showed similar peaks to the previous study of MH spectrogram, as the peaks at 3367 cm ^-1^, 3300 cm ^-1^ and 1580 cm ^-1^ associated with the distinct group of N-H asymmetric stretching, N-H symmetric stretching and N-H bending, respectively. ^39^ Also, the Na alginate spectrogram showed a peak at 1619 cm ^-1^, which is the position of the carbonyl group of Na alginate beads. ^40^ Figure 3(B) demonstrates beads F4, F6 and F7 spectrograms emphasizing a peak deviation associated with the carbonyl group to 1629 cm ^-1^, as this might refer to Na alginate molecules binding with calcium ions that helped to arrange and build the beads. ^41^ The N-H asymmetric stretching, N-H symmetric at 3367 cm ^−1^ and 3300 cm ^−1^ overlaid with OH region of the hydroxyl group of sodium alginate as it hard to tell if physical interaction happened in the spectrograms of F4, F6 and F7 related to the mentioned groups; however, MH might physically interact at N-H group as there is a shift of the band at 1580 cm ^−1^ to a lower region. Although it is not inconclusive, it can indicate the hydrogen bondings.

### Metformin absorption *in silico* model

**Figure 4.**
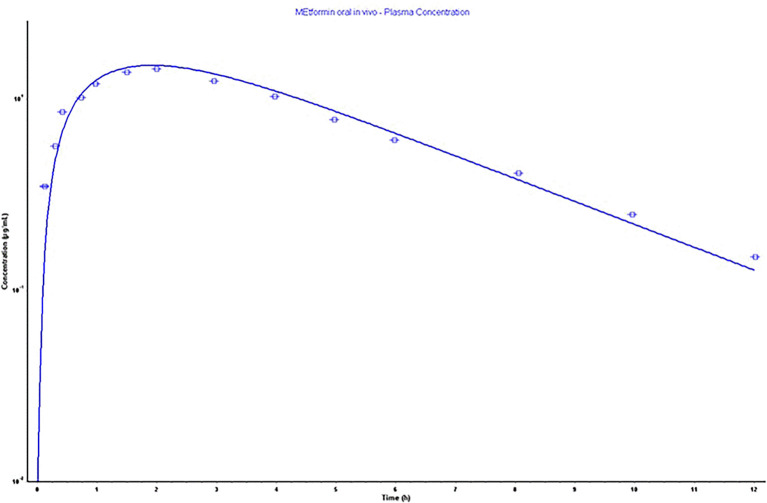
500 mg oral immediate tablet physiological model as the bold line represents calculated and the dotted line represents the observed values.

The parameters as an outcome of the *in silico* simulation that assisted in building a physiological model relied on the physicochemical and pharmacokinetic information are presented in Table 5. Also, the constructed model parameters Cmax, Tmax, AUC 0-inf, and AUC 0-t were validated, as screened in Figure 4, depending on the error percentage of observed and calculated data. The acceptance of %PE is valid as the calculated values do not double the observed values or the fold error value is not doubled. ^42^ The model showed a very acceptable error in percentage, as shown clearly in Table 5.

**Table 5.** The predicting and observed values of metformin pharmacokinetic parameters of Gastroplus ^®^ for 500 mg oral immediate-release tablet.

Pharmacokinetic parameters	Observed	Calculated	% PE
Cmax ^a^ (μg/mL)	1.44	1.5021	-4.312
Tmax ^b^ (h)	2	1.92	4
AUC ^c^ 0-∞ (μg-h/mL)	8.8805	8.9842	-1.16
AUC 0-t (μg-h/mL)	8.2756	8.5212	-2.9

^a^ Cmax denotes maximum serum concentration.

^b^ Tmax, the time required to reach maximum concentration.

^c^ AUC donates area under the curve, the negative values of %PE mean the predicted are >than observed.

**Figure 5.**
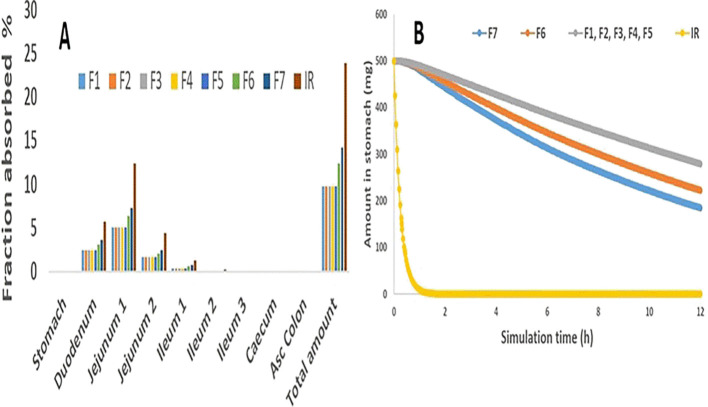
Predicting data, Figure A: The regional absorption of the 500 mg MH selected gastroretentive multiple unit floating beads; Figure B: The MH amount retained in the stomach for 12 h, as IR represents the immediate release tablet and the blue curve denotes F1, F2, F3, F4, and F5 in Figure B.

Moreover, Figure 5A demonstrates that absorption does not take place in the stomach, whereas the maximum MH absorption occurred in jejeunum 1, followed by the duodenum and jejeunum 2, and then almost nowhere else. Similarly, as the prediction results revealed, the MH multiple unit floating gastroretentive drug delivery system exhibited no stomach absorption site. ^11^ Also, the fraction absorbed (total availability) was 23.9, of Metformin IR of the model, as seen in Figure 5A, which was close to 27%, the predicted fraction absorbed that was found by the Dahan study. ^16^ Consequently, Figure 5B indicates that F1 to F5 beads formulas provided high stomach amounts following their floating duration and showed a gradual with slight differences decrease in MH amount within 12 hrs representing its floating duration, while F7 showed a drastic decrease in the stomach amount of MH within the first hour of simulation time. Additionally, F6 presented a gradual reduction in the amount of MH in the stomach within simulation time. These outcomes referred that these gastroretentive multiple unit floating beads play an essential role in restricting the MH release rate in the site-specific region, which in turn guarantees the release of MH into the appropriate absorption site and, thus, may improve the bioavailability.

**Figure 6.**
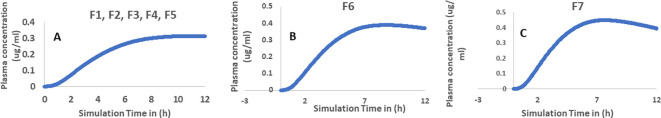
Predicted plasma concentration-time curves of MH multiple unit beads.

In the same figure, for comparison purposes, immediate-release kinetic was taken from an immediate-release tablet of 500 mg of MH that showed (rapid MH decline). ^43^ The simulating curves of plasma concentration-time as in Figure 6 parameters reveal that F1-F5 exhibit low Cmax with non-declining curves, thus referring to the gastroretentive property of slow-release MH multiple unit floating beads. At the same time, F6 and F7 showed a higher Cmax, as F7 showed a decline in the curve; this might be attributed to the total released amount of MH in the stomach. However, the floating period was persistent during the *in vitro* release study. Interestingly, the US Food and Drug Administration revealed the pharmacokinetics parameters of a 500 mg extended-release tablet, as its Cmax was 0.6 μg/ml, which was close to the Cmax of 0.449 μg/ml of F7. ^44^ Taking this into account, the MH simulation helped to decide the better formulation of floating beads that achieved the aim of this study.

### Future perspective

During drug development, the bridge between the drug characteristics and *in vivo* behaviour of pharmacokinetics can be achieved by the vital *in silico* tool (PBPK). In recent decades, the *in silico* tool has been widely applied to different drug delivery systems and different routes of administration, including oral, inhaled, transdermal, ophthalmic, and complex injectable products, for small drug molecules to more complex therapeutics. The results of the simulation have provided important insights into the PK behaviours of new dosage forms, which strongly support drug regulation. ^45^ In the current research the future perspective involves optimization and development of the prepared dosage form, taking into consideration the specific physiological parameters for instance, age, weight and renal function, these factors may require dose adjustment or altered regimens, especially in certain population including elderly, renal and hepatic impatient. In addition, the physiology changes related to diabetes mellites such as the gastrointestinal motility and pH changes, or cancer-associated alteration in pH due to the tumour microenvironment, or changes related to gastrectomy or ulcer affecting drug solubility and absorption process. Consequently, these considerations could be integrated to develop personalized PBPK and enhance the predictive capability of the models, leading to more precise simulations for drug absorption and disposition, correspondingly paving the way for personalised medicine with patient-specific modelling, eventually enhancing therapeutic efficacy and safety. Furthermore, The Gastroplus may take the lead toward enhanced IVIV correlation by establishing robust IVIVC models that aid in the transition from laboratory findings to clinical applications reducing the dependence on prevalent *in vivo* studies.

### Limitation

More accurate results are expected to be produced through the use of the PBPK modelling in drug delivery at high speed. Though recently, many limitations have impeded this goal. The PBPK will not catch the interindividual variability due to genetic differences, comorbidities, or concurrent medications which have an impact on drug absorption and metabolism.

## Conclusion

This study aimed to develop a method for MH to create multi-unit beads utilized for gastroretentive purposes. Numerous tests were applied, including percentage yield, entrapment efficiency percent, floating property, and the *in vitro* release study. All prepared beads floated for their corresponding release time and showed different release patterns. Gastroplus ^®^, a software, was used to acquire fruitful models of MH. *In silico* results based on *in vitro* release and floating property demonstrated that F1-F5 beads with high stomach MH amount supported the progressive reduction in MH released amount within simulation time, whereas F6 showed a rapid decline in the stomach amount corresponding to the faster MH release. The interesting formulation F7 exhibited a gradual decrease in MH amount and established a close Cmax to the 500 mg extended-release tablet. Furthermore, the Gastroplus ^®^ software simulation found the highest MH absorption location in jejunum 1, followed by the duodenum. The utilization of GastroPlus ^®^ in PBPK modelling provides an influential platform for the design and optimization of bead-based drug delivery systems, paving the way for more effective and personalized therapeutic strategies and enhancement in IVIV correlation.

## Contributor role

Conceptualization: Sura Zuhair Mahmood, Nora Zawar Yousif and Masar Basim Mohsin Mohamed

Data Curation: Sura Zuhair Mahmood, Nora Zawar Yousif and Masar Basim Mohsin Mohamed

Formal Analysis: Nora Zawar Yousif and Masar Basim Mohsin Mohamed

Funding Acquisition: Sura Zuhair Mahmood, Nora Zawar Yousif and Masar Basim Mohsin Mohamed

Investigation: Sura Zuhair Mahmood, Nora Zawar Yousif and Masar Basim Mohsin Mohamed

Methodology: Sura Zuhair Mahmood

Project Administration: Sura Zuhair Mahmood, Nora Zawar Yousif and Masar Basim Mohsin Mohamed

Resources: Sura Zuhair Mahmood, Nora Zawar Yousif and Masar Basim Mohsin Mohamed

Software: Masar Basim Mohsin Mohamed

Supervision: Masar Basim Mohsin Mohamed

Validation: Masar Basim Mohsin Mohamed

Visualization: Sura Zuhair Mahmood, Nora Zawar Yousif and Masar Basim Mohsin Mohamed

Writing – Nora Zawar Yousif and Masar Basim Mohsin Mohamed

Writing – Review & Editing: Nora Zawar Yousif and

## Ethics and consent

Not applicable.

## Data Availability

Zenodo:Forecasting in vivo pharmacokinetics of metformin HCl Floatin g beads using Gastroplus ^®^ PBPK ( https://zenodo.org/records/14197021). ^46^ This project contains the following files:
•
FTIR of Metformin.xlsx
•
the last one inshallah.xlsx
•
the raw data of release and gastroplus.xlsx Zenodo
• The raw data of amount in stomach https://zenodo.org/records/15692145 ^47^
• FTIR Metformin figure https://zenodo.org/records/15692151 ^48^
• Plasma time figure formulation https://zenodo.org/records/15692186 ^49^
• Regional Absorption https://zenodo.org/records/15692198 ^50^
• Release Metformin figure https://zenodo.org/records/15692204 ^51^
• Metformin in vivo Data https://zenodo.org/records/15692171 ^52^ Data are available under the terms of the Creative Commons Attribution 4.0 International license (CC-BY 4.0).
