# Supplementary material for: Forecasting in vivo pharmacokinetics of metformin HCl floating beads using Gastroplus® PBPK
Source: F1000Res. 2025 Sep 9;14:140. Originally published 2025 Jan 28. [Version 2] doi: 10.12688/f1000research.159438.2 (PMC12485330; doi:10.12688/f1000research.159438.2)
Supplement: Peer review history

# Reviewer report: Reviewer response for version 2

**DOI:** 10.5256/f1000research.183289.r414010

**Published:** 2025-09-30

**Authors:** Sadia Rehman, Murryam Nawaz

**Affiliations:** 1 Pharmacy, Riphah Interntional University, Islamabad, Islamabad Capital Territory, Pakistan; 2 Riphah International University, Islamabad, Pakistan

Summary of the Article:

 The manuscript reports the fabrication of metformin hydrochloride (MH) floating beads using an ionotropic gelation technique with alginate and cross-linking via CaCl₂. Seven formulations were prepared and evaluated for yield, entrapment efficiency, floating behavior, and in vitro drug release. The optimized formulation was characterized by FTIR, FESEM, and swelling studies. To translate in vitro findings into in vivo performance, a physiologically based pharmacokinetic (PBPK) model was constructed using GastroPlus®. The model incorporated known metformin transporter interactions (OCT1/2, MATE1/2, PMAT) while excluding OCT3. Simulation outcomes were compared to reported pharmacokinetic profiles of immediate-release tablets, and the authors suggest that the floating beads offer enhanced gastro-retentive behavior and absorption. The manuscript also includes a limitation and future perspective section, highlighting personalized PBPK approaches.

 Overall Assessment:

 The study addresses a clinically relevant problem — improving the delivery of MH, a first-line drug for type II diabetes, through gastroretentive floating beads combined with PBPK modeling. The topic is interesting and potentially useful for pharmaceutical formulation and predictive modeling research.

 However, the presentation lacks clarity, the statistical analysis is weak, and the PBPK model validation is insufficient. While the manuscript has merit, it requires major revision before it can be considered scientifically sound.

 Major Comments:

1.
  1. Entrapment efficiency, yield, and in vitro release data are presented as mean ± SD, but no statistical comparisons (ANOVA, t-tests) are performed between formulations. Without significance testing, it is unclear whether differences between formulations are meaningful.
  2. The PBPK model is validated against only one dataset (500 mg IR tablets).
  3. Sensitivity analysis on transporter inclusion/exclusion (especially OCT3) should be considered or at least justified more thoroughly.
  4. Table 2 (formulation composition) appears incomplete in the submitted version. Full details of polymer concentrations, curing times, and number of replicates should be included for reproducibility. Raw dissolution data with error bars should be presented.
  5. The conclusion that PBPK modeling “confirms” enhanced absorption of MH beads is overstated whereas, the evidence only suggests a potential improvement. Authors should moderate their claims and align conclusions more closely with the presented data.
  6. Abstract should be more concise — clearly state aim, methods, key results, and conclusion.
  7. Literature review needs updating with recent studies (2019–2024) on metformin gastroretentive systems and PBPK applications.

Checklist Answers with Justifications:

1. Is the work clearly and accurately presented and does it cite the current literature?
  Partly – The structure is clear, but language issues obscure meaning. References are somewhat outdated; more recent citations are needed.
2. Is the study design appropriate and is the work technically sound?
  Partly – The bead fabrication and PBPK approach are appropriate. However, the PBPK validation is weak and statistical rigor is lacking.
3. Are sufficient details of methods and analysis provided to allow replication by others?
  Partly – Important details (full formulation table, replicate numbers, raw data) are missing, limiting reproducibility.
4. Is the statistical analysis and its interpretation appropriate?
  Partly – Basic descriptive statistics are given, but no comparative statistical analysis has been applied.
5. Are all the source data underlying the results available to ensure full reproducibility?
  Partly – Key raw data and details are not provided in sufficient depth.
6. Are the conclusions drawn adequately supported by the results?
  Partly – Conclusions are generally in line with results, but claims of enhanced absorption should be toned down given the limited validation.

Is the work clearly and accurately presented and does it cite the current literature?

Partly

If applicable, is the statistical analysis and its interpretation appropriate?

Partly

Are all the source data underlying the results available to ensure full reproducibility?

Partly

Is the study design appropriate and is the work technically sound?

Partly

Are the conclusions drawn adequately supported by the results?

Partly

Are sufficient details of methods and analysis provided to allow replication by others?

Partly

Reviewer Expertise:

Pharmaceutical technology, formulation development, drug delivery systems, emulsions, suspensions, hydrogels,  nanogels, nanoparticles.

We confirm that we have read this submission and believe that we have an appropriate level of expertise to confirm that it is of an acceptable scientific standard, however we have significant reservations, as outlined above.

# Reviewer report: Reviewer response for version 2

**DOI:** 10.5256/f1000research.183289.r412668

**Published:** 2025-09-17

**Authors:** Sagar Salave

**Affiliations:** 1 National Institute of Pharmaceutical Education and Research, Ahmedabad, India; 2 Pharmaceutical Chemistry, The University of Kansas (Ringgold ID: 4202), Lawrence, Kansas, USA

Authors have made significant changes in the revised manuscript

Is the work clearly and accurately presented and does it cite the current literature?

Yes

If applicable, is the statistical analysis and its interpretation appropriate?

Partly

Are all the source data underlying the results available to ensure full reproducibility?

Yes

Is the study design appropriate and is the work technically sound?

Partly

Are the conclusions drawn adequately supported by the results?

Yes

Are sufficient details of methods and analysis provided to allow replication by others?

Partly

Reviewer Expertise:

Drug delivery, formulation development, Nanomedicine, vaccines

I confirm that I have read this submission and believe that I have an appropriate level of expertise to confirm that it is of an acceptable scientific standard.

# Reviewer report: Reviewer response for version 1

**DOI:** 10.5256/f1000research.175161.r364759

**Published:** 2025-04-15

**Authors:** Anuj Garg

**Affiliations:** 1 Institute of Pharmaceutical Research, GLA University, Mathura, Uttar Pradesh, India

In the present research work, the authors have developed floating beads containing metformin and forecast the plasma concentration using Gastroplus software on the basis of previous in vivo results and in vitro results performed in the present study.

 The author describes the methodology appropriately and well written in the manuscript. However, the references are not upto date and most of the cited references are before 2020. 

 Moreover, the objective of this study was to develop floating beads which seems to be very old method and various studies have been already published for the same objective to formulate floating beads. 

 One of the paper is as follow:

 Nayak, A., Jain, S. K., & Pandey, R. S. (2011 [Ref-1]). Controlling release of metformin HCl through incorporation into stomach specific floating alginate beads. *Molecular pharmaceutics*, *8*(6), 2273-2281.

 The interpretation of results should be discussed in depth to explain the possible reason behind the results.

Is the work clearly and accurately presented and does it cite the current literature?

Partly

If applicable, is the statistical analysis and its interpretation appropriate?

Partly

Are all the source data underlying the results available to ensure full reproducibility?

No source data required

Is the study design appropriate and is the work technically sound?

Yes

Are the conclusions drawn adequately supported by the results?

Yes

Are sufficient details of methods and analysis provided to allow replication by others?

Yes

Reviewer Expertise:

Drug delivery, Pharmacokinetics

I confirm that I have read this submission and believe that I have an appropriate level of expertise to confirm that it is of an acceptable scientific standard, however I have significant reservations, as outlined above.

## References

[1] : Controlling release of metformin HCl through incorporation into stomach specific floating alginate beads. *Mol Pharm* .2011;8(6) : 10.1021/mp2001395 2273-81 10.1021/mp2001395 22017707

# Response

**Published:** 2025-06-16

**Authors:** Sura Zuhair Mahmood

**Affiliations:** Pharmaceutics Department, Pharmacy College, Mustansiriyah University, Baghdad, Iraq1, Iraq

In the present research work, the authors have developed floating beads containing metformin and forecast the plasma concentration using Gastroplus software on the basis of previous in vivo results and in vitro results performed in the present study.

 The author describes the methodology appropriately and well written in the manuscript. However, the references are not up-to-date and most of the cited references are before 2020. Moreover, the objective of this study was to develop floating beads which seems to be very old method and various studies have been already published for the same objective to formulate floating beads. 

**Thank you for your valuable comments and observations. We have updated most of the references to ensure they are more current. While we acknowledge that the method used is established and considered an old technique, we believe it remains highly reproducible and capable of yielding reliable results. Furthermore, previous studies have not addressed the prediction of in vivo pharmacokinetics based on in vitro release data using this specific approach, which we believe adds novelty to our work, and this was referred to in the introduction section**

 One of the paper is as follows:

 Nayak, A., Jain, S. K., & Pandey, R. S. (2011 [Ref-1]). Controlling release of metformin HCl through incorporation into stomach specific floating alginate beads. *Molecular pharmaceutics*, *8*(6), 2273-2281.

**Kindly refer to the introduction and reference sections, which were changed to more recent ones as per your request**

 The interpretation of results should be discussed in depth to explain the possible reasons behind the results 

**Thank you for pointing this out. The revised version now includes a more detailed explanation** **and is highlighted within the result section.**

# Reviewer report: Reviewer response for version 1

**DOI:** 10.5256/f1000research.175161.r364754

**Published:** 2025-02-18

**Authors:** Sagar Salave

**Affiliations:** 1 National Institute of Pharmaceutical Education and Research, Ahmedabad, India; 2 Pharmaceutical Chemistry, The University of Kansas (Ringgold ID: 4202), Lawrence, Kansas, USA

Authors need to revise the manuscript thoroughly as per the below comments: 

 Major comments: 

 1. Improve the abstract 

 2. Improve the introduction section, and discuss recent studies with limitations instead of just an overview for better comparison

 3. The author needs to discuss results in detail rather than just observations 

 Minor comments:

 1. Correct annotations throughout the manuscript (eg. Figures)

 2. Figure 6, please correct Y-axis label 

 3. Include scale bar in SEM images

 4.. Write a future perspective of the current study 

 5.. What are the limitations of this study

Is the work clearly and accurately presented and does it cite the current literature?

Yes

If applicable, is the statistical analysis and its interpretation appropriate?

Partly

Are all the source data underlying the results available to ensure full reproducibility?

Yes

Is the study design appropriate and is the work technically sound?

Partly

Are the conclusions drawn adequately supported by the results?

Yes

Are sufficient details of methods and analysis provided to allow replication by others?

Partly

Reviewer Expertise:

Drug delivery, formulation development, Nanomedicine, vaccines

I confirm that I have read this submission and believe that I have an appropriate level of expertise to state that I do not consider it to be of an acceptable scientific standard, for reasons outlined above.

# Response

**Published:** 2025-06-16

**Authors:** Sura Zuhair Mahmood

**Affiliations:** Pharmaceutics Department, Pharmacy College, Mustansiriyah University, Baghdad, Iraq1, Iraq

1. Improve the abstract 

**The aim of the abstract has been revised to improve clarity.**

 2. Improve the introduction section, and discuss recent studies with limitations instead of just an overview for better comparison

**Thank you for your careful reading and valuable feedback.** **Yes, the highlighted parts of the introduction were improved by changing the aim of the study and referring to the limitations in the other previous studies regarding metformin HCl formulations.**

 3. The author needs to discuss results in detail rather than just observations 

**Yes, this change has been implemented and highlighted in the results of the revised version.**

 Minor comments:

 1. Correct annotations throughout the manuscript (eg. Figures)

**The annotations have been revised accordingly, especially in Figures 2  and 4**

 2. Figure 6, please correct the Y-axis label 

**Yes, it is done**

 3. Include a scale bar in SEM images

**Yes,  included in the image**

 4. Write a future perspective of the current study 

**Done, and it is highlighted in the revised version**

 5. What are the limitations of this study

**The limitation was written at the end of the revised version**

 1. Improve the abstract 

**The aim of the abstract has been revised to improve clarity.**

 2. Improve the introduction section, and discuss recent studies with limitations instead of just an overview for better comparison

**Thank you for your careful reading and valuable feedback.** **Yes, the highlighted parts of the introduction were improved by changing the aim of the study and referring to the limitations in previous studies regarding metformin HCl formulations.**

 3. The author needs to discuss results in detail rather than just observations 

**Yes, this change has been implemented and highlighted in the result of the revised version.**

 Minor comments:

 1. Correct annotations throughout the manuscript (eg. Figures)

**The annotations have been revised accordingly, especially in Figures 2  and 4**

 2. Figure 6, please correct the Y-axis label 

**Yes, it is done**

 3. Include a scale bar in SEM images

**Yes,  included in the image**

 4. Write a future perspective of the current study 

**Done, and it is highlighted after  the end of the revised version**

 5. What are the limitations of this study

**The limitations were written at the end of the revised version**
